# Whole Genome Sequence of an Edible and Potential Medicinal Fungus, *Cordyceps guangdongensis*

**DOI:** 10.1534/g3.118.200287

**Published:** 2018-04-17

**Authors:** Chenghua Zhang, Wangqiu Deng, Wenjuan Yan, Taihui Li

**Affiliations:** State Key Laboratory of Applied Microbiology Southern China, Guangdong Provincial Key Laboratory of Microbial Culture Collection and Application, Guangdong Open Laboratory of Applied Microbiology, Guangdong Institute of Microbiology, Guangzhou, 510070, China

**Keywords:** *Cordyceps*, chromosome, transporters, transcription factors, Genome Report

## Abstract

*Cordyceps guangdongensis* is an edible fungus which was approved as a novel food by the Chinese Ministry of Public Health in 2013. It also has a broad prospect of application in pharmaceutical industries, with many medicinal activities. In this study, the whole genome of *C. guangdongensis* GD15, a single spore isolate from a wild strain, was sequenced and assembled with Illumina and PacBio sequencing technology. The generated genome is 29.05 Mb in size, comprising nine scaffolds with an average GC content of 57.01%. It is predicted to contain a total of 9150 protein-coding genes. Sequence identification and comparative analysis indicated that the assembled scaffolds contained two complete chromosomes and four single-end chromosomes, showing a high level assembly. Gene annotation revealed a diversity of transposons that could contribute to the genome size and evolution. Besides, approximately 15.57% and 12.01% genes involved in metabolic processes were annotated by KEGG and COG respectively. Genes belonging to CAZymes accounted for 3.15% of the total genes. In addition, 435 transcription factors, involved in various biological processes, were identified. Among the identified transcription factors, the fungal transcription regulatory proteins (18.39%) and fungal-specific transcription factors (19.77%) represented the two largest classes of transcription factors. This genomic resource provided a new insight into better understanding the relevance of phenotypic characters and genetic mechanisms in *C. guangdongensis*.

*Cordyceps guangdongensis* T. H. Li, Q. Y. Lin & B. Song was discovered in Southern China (Lin *et al.* 2008), and has been successfully cultivated (Lin *et al.* 2010). Its fruiting body is nontoxic, and was approved as the second novel food of *Cordyceps* species by the Ministry of Public Health of China in 2013. This fungus is rich in nutrients and bioactive compounds, such as cordycepic acid, adenosine and polysaccharides (Lin *et al.* 2009; 2010); these contents are similar to those of the traditional Chinese invigorant, *Ophiocordyceps sinensis* (=*Cordyceps sinensis*) (Lin *et al.* 2009). Previous research by the authors’ group indicated that the fruiting bodies of *C. guangdongensis* possessed various therapeutic properties, including antioxidant activity (Zeng *et al.* 2009), longevity-increasing activity (Yan *et al.* 2011), anti-fatigue effect (Yan *et al.* 2013), curative effect on chronic renal failure (Yan *et al.* 2012), and anti-inflammatory effect (Yan *et al.* 2014). These active effects provided great potential for its application in food and medicinal industries. Therefore, it is a matter of cardinal significance to further understand the fruiting body development and metabolic mechanisms of *C. guangdongensis*, as well as its evolutionary relationship with other related species.

In recent years, whole genome sequencing (WGS) has been widely used to analyze the relevance of phenotypic characters and genetic mechanisms. The rapid development of advanced sequencing techniques and bioinformatic methods makes it more convenient to further explore the mechanisms of fungal development, metabolism, systematic taxonomy, and evolution at the molecular level. To date, numerous genomes of fungi within the order Hypocreales have been published in the Ensembl fungus database (http://fungi.ensembl.org/index.html). Based on the available genome sequences, researchers not only ascertained evolutionary relationship of many fungi (Zheng *et al.* 2011; Bushley *et al.* 2013; Xia *et al.* 2017), but also identified almost 40 medically active product producing gene clusters in different *Cordyceps* species, such as cyclosporine, oosporein, beauvericin, efrapeptins, 2-pyridone alkaloids, equisetin, emericellamide, tolypin (Zheng *et al.* 2011; Bushley *et al.* 2013; Quandt *et al.* 2015; Kramer and Nodwell 2017; Lu *et al.* 2017). Meanwhile, various transcription factors (TFs), including bZIP TFs, zinc finger TFs and fungal-specific TFs, were proven to be involved in fruiting body development by transcriptome analysis on the basis of genome sequences (Zheng *et al.* 2011; Yin *et al.* 2012; Yang *et al.* 2016). However, the whole genome sequence for *C. guangdongensis* is still lacking.

In order to acquire abundant molecular information to effectively explore the genetic characteristics of *C. guangdongensis*, the whole genome of *C. guangdongensis* was sequenced for the first time in this study. At the assembly level, the types of transposable elements and transcriptional factors (TFs) were further analyzed. This genomic resource provids a new insight to better understand the relevance of phenotypic characters and genetic mechanisms in *C. guangdongensis*.

## Material and Methods

### Fungal strains and DNA extraction

The sample used for the whole genome sequencing and assembly was isolated from the strain GDGM30035 (wild fruiting bodies of *C. guangdongensis*). The strain was cultured on PDA medium at 23 ± 1° for four weeks. Aqueous suspensions of fungal spores were prepared by pouring sterile distilled water onto the sporulated cultures and gently scrubbing the agar surface. The spore suspension was collected by passing the aqueous fungal suspensions through four layers of sterile cheesecloth to remove mycelial fragments. The spore suspension was diluted to 1×10^3^ conidia ml/L using a hemocytometer, and was coated on the PDA medium covered with cellophane. A single colony (*C. guangdongensis* GD15) was transferred onto a new PDA medium and was cultured three times. For DNA isolation, the strain was cultured on a PDA medium, which was covered with cellophane in advance. Genomic DNA from a 7-day-old fungal colony was extracted using CTAB-based extraction buffer (Watanabe *et al.* 2010). The DNA concentration was determined using UV-Vis spectrophotometer (BioSpec-nano), the integrity of the DNA was detected using 0.8% agarose gel, and the purity of the DNA was analyzed with PCR using 16S rDNA primers.

### Genome sequencing and assembly

The genome of *C. guangdongensis* GD15 was sequenced at the Beijing Genomics Institute at Shenzhen with the hybrid of Illumina HiSequation 2000 and the PacBio sequencing platform. The PacBio sequencing approach could provide previously unprecedented sequencing read lengths (>2kb), and get better sequencing depth. The next generation sequencing approach has also been widely used in various species. It has lower mismatch rate, with shorter sequencing read lengths. To cater for mismatch rate and get better sequencing read lengths, we combined the next generation sequencing approach (Illumina HiSeq) and the PacBio sequencing approach for the genome sequencing. DNA libraries with 500 bp inserts were constructed and sequenced with the Illumina HiSeq2000 Genome Analyzer. Long insert SMRTbell template libraries were prepared according to PacBio protocols. The unqualified raw reads obtained by PacBio were filtered out, the subreads (≥1000bp) were corrected by Proovread 2.12 (https://github.com/BioInf-Wuerzburg/proovread) (Hackl *et al.* 2014), and were initially assembled by SMRT Analysis v.2.3.0 (Chin *et al.* 2013). The preliminary assembly results were further corrected using small Illumina reads by GATK v1.6-13 (http://www.broadinstitute.org/gatk/) (De Summa *et al.* 2017), and the scaffolds were assembled and optimized using long Illumina reads by SSPACE Basic v2.0 (http://www.baseclear.com/genomics/bioinformatics/basetools/SSPACE) and PBJelly2 v15.8.24 (https://sourceforge.net/projects/pb-jelly/) (English *et al.* 2012). The completeness of this assembly was assessed using the BUSCO analysis described by Simão *et al.* (2015).

### Genome components analysis

The characteristic telomeric repeats (TTAGGG/CCCTAA) were searched for on both ends of each scaffold within 100bp length. Repetitive elements included tandem repeats and transposable elements (TEs). Tandem repeats were searched for in all scaffolds with Tandem Repeats Finder (TRF 4.04), as described by Benson (1999). TEs annotation was performed with RepeatMasker 4.06 (Smit *et al.* 2014) based on the Repbase database (http://www.girinst.org/repbase/). The tRNAs were predicted using tRNAscan-SE 1.3.1 (Lowe and Eddy 1997), rRNAs were identified using RNAmmer 1.2 (Lagesen *et al.* 2007), and sRNA were predicted with Infernal based on the Rfam database (Gardner *et al.* 2009). Genes were annotated based on sequence homology and *de novo* gene predictions. The homology approach was based on the reference genomes downloaded from EnsemblFungi (http://fungi.ensembl.org/index.html) including the protein sequences of *C. militaris*, *O. sinensis* and *Cordyceps ophioglossoides* (=*Tolypocladium ophioglossoides*). The *de novo* gene predictions were performed with Genemark-ES 4.21 (Ter-Hovhannisyan *et al.* 2008).

### Functional annotation

Structural and functional annotations of genes were performed according to various databases of ARDB (Antibiotic Resistance Genes Database) (Liu and Pop 2009), CAZymes (Carbohydrate-Active enZYmes Database) (Cantarel *et al.* 2009), COG (Cluster of Orthologous Groups) (Tatusov *et al.* 2003), GO (Gene Ontology) (Ashburner *et al.* 2000), KEGG (Kyoto Encyclopedia of Genes and Genomes) (Kanehisa *et al.* 2006), NR (Non-Redundant Protein Database) (Yu and Zhang 2013), P450 (Magrane *et al.* 2011), PHI (Pathogen Host Interactions) (Torto-Alalibo *et al.* 2009), SwissProt (Magrane *et al.* 2011), T3SS (Type III Secretion System Effector protein) (Vargas *et al.* 2012), TrEMBL (O’Donovan *et al.* 2002), VFDB (Virulence Factors of Pathogenic Bacteria) (Chen *et al.* 2016), IPR (InterPro Protein Families Database) (Mitchell *et al.* 2015), KOG (Eukaryotic Orthologous Groups) (Tatusov *et al.* 2003), and NOG (Non-supervised Orthologous Groups) (Huerta-Cepas *et al.* 2016). Transcription factors were annotated according to their InterPro IDs in the Fungal Transcription Factor Database (Wilson *et al.* 2008).

### Data availability

The genome sequencing project has been deposited at GenBank under the accession number NRQP00000000. The BioProject designation for this project is PRJNA399600. Figure S1 shows the length and quality distributions of PacBio reads. Figure S2 shows the BUSCO analysis of the completeness of the assembly results. Figure S3 shows the distribution of gene length predicted in the *C. guangdongensis* genome. Table S1 shows the genome sequences used to analyze the chromosome. Table S2 shows the genome annotation of proteins in *C. guangdongensis* genome. Table S3 shows the transposable element classification in *C. guangdongensis*. Supplemental material available at Figshare: https://figshare.com/s/6ad20d1f75328ca704ca.

## Results and Discussion

### Whole-genome assembly

**Figure 1:**
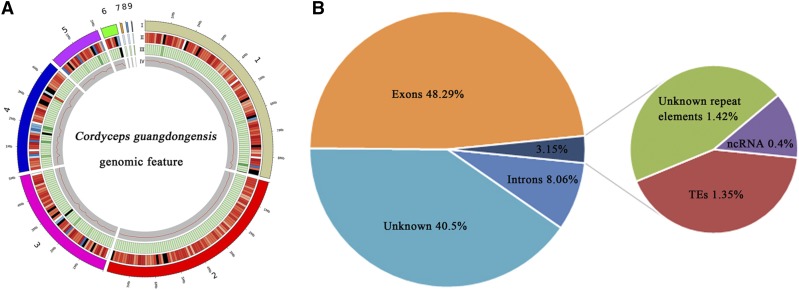
General genomic features of *Cordyceps guangdongensis*. A, I, scaffolds, the different colors represented different scaffolds; II, gene density, represented as the number of genes per 100 kb, increased in color intensity from light blue, to dark blue, dark, dark red, and light red. The density of non-coding RNA increased in color intensity from dark blue, to light blue, white, light red, and dark red; III, percentage of coverage of repetitive sequences, increased in color intensity from light green, to dark green, dark, dark red, and light red; IV, GC content estimated by the percentage of G + C in 100 kb. B, Genomic element density including genic and nongenic features of the overall genome assembly length including 40.5% non-annotated sequences.

A total of 3,926,378,523 reads representing a cumulative size of 3.926 Gb were generated, including 13,392,532 and 3,912,985,991 reads from Illumina and PacBio sequencing platforms, respectively. The PacBio sequencing results showed high quality polymerase reads and subreads (Figure S1). After filtering out the low quality reads, a total of 3,484,503,143 reads were assembled into nine scaffolds with N50 of 7.88 Mb from ∼183 average coverage. In addition, a total length of 29.05 Mb with a 57.01% GC content was obtained (Figure 1A). Based on the Illumina sequencing data, the predicted genome size by K-mer analysis was 31.58Mb; the total size of the combined assembly closely matched this estimated size (91.98%). Compared to the previously reported draft genomes listed in Table 1. The completeness of the assembly results was evaluated by comparing with the BUSCO set of 1315 fungal orthologs. According to the results, a total of 1295 set appeared complete in the *C. guangdongensis* gene sets; this indicated an estimated completeness of 98.5%, with only 0.99% missing (Figure S2). These results indicated that our assembly is relatively contiguous.

**Table 1:** Assembly summary statistics of *Cordyceps guangdongensis* GD15 compared to other *Cordyceps* genomes

Species	*C. guangdongensis*	*C. militaris* CM01	*O. sinensis* CO18	*C. cicadae* CCAD02
NCBI Bio Project	NRQP00000000	AEVU00000000	ANOV00000000	MWMN00000000
Assembly size (Mb)	29.0	32.2	78.5	33.9
Coverage fold	183x	147x	241x	80x
No. of Scaffold	9	32	10603	599(>1 kb)
N50	7.88 Mb	0.11 Mb	5.39 kb	0.21 Mb
GC%	57.0	51.4	46.1	53.0
Repeat content (%)	2.77	3.04	37.98	3.19
Gene density (genes per Mb)	315	301	87	286

### Chromosome analysis

Sequence analysis of telomeric repeats was used to estimate the number of chromosomes in the *C. guangdongensis* genome according to the method described by Zheng *et al.* (2011). The characteristic telomeric repeats (TTAGGG/CCCTAA)_n_ were found at either 5 ʹ or 3 ʹ terminal of six scaffolds, of which the telomeric repeats were found at both ends of scaffolds one and three, suggesting that the two scaffolds are complete chromosomes. The lengths of the two complete chromosomes were about 8.81 Mba and 5.00 Mba, respectively. Single-ended telomeric repeats were found at four scaffolds, including the start of scaffolds four and six, the ends of scaffolds two and five, suggesting that these four scaffolds extended to the telomeres. The lengths of the four candidate scaffolds were about 7.88, 4.50, 2.05, and 0.61 Mba, respectively. The remaining three scaffolds contained no telomeric repeats, possibly due to incompleteness of the scaffold sequence data (Table 2, Table S1). Furthermore, on scaffold seven, 14 genes were identified which belonged to the core genes of mitochondrial genome, indicating that this scaffold represents mitochondrial genome sequence. Previous researches showed that the haploid genome of *C. militaris* contains seven chromosomes (Kramer and Nodwell 2017), and *Cordyceps subsessilis* (=*Tolypocladium inflatum*) also contains seven chromosomes (Stimberg *et al.* 1992). Taking into consideration the chromosome number of these related species and the present telomeric repeats analysis, it was inferred that *C. guangdongensis* may also possibly contain seven chromosomes. This hypothesis should be further proved with karyotype analysis.

**Table 2:** Chromosome analysis of *Cordyceps guangdongensis* GD15 genomic sequence

Scaffold	Size (bp)	Start Telomere	End Telomere	Judge	chromosome
Scaffold1	8,817,043	CCCTAA	TTAGGG	double-end	Complete chromosome
Scaffold2	7,881,840	No	TTAGGG	single-ended	Chromosome fragment
Scaffold3	5,000,199	CCCTAA	TTAGGG	double-end	Complete chromosome
Scaffold4	4,508,454	CCCTAA	No	single-ended	Chromosome fragment
Scaffold5	2,058,248	No	TTAGGG	single-ended	Chromosome fragment
Scaffold6	614,660	CCCTAA	No	single-ended	Chromosome fragment
Scaffold7	75,887	No	No	No	Mitochondrial genome
Scaffold8	68,140	No	No	No	Fragment
Scaffold9	31,250	No	No	No	Fragment

### Genome features and annotation

As shown in Table 3, a total of 9150 protein-coding genes were predicted in the genome, including 31 rRNA, 111 tRNA, 121 sRNA, 25 snRNA and 26 miRNA. The cumulative length of the total number of genes accounted for 56.35% of the whole genome sequence length, and the lengths of most genes were in the range of 200-5000bp (Figure S3). There was a large proportion of exons (48.29%), with a maximum number of 29,548, and the number of introns was 20,398, with a total length of 2.34 Mba (8.06%). The total number of ncRNA was 314, representing 0.4% of the genome assembly; this suggested that ncRNA formed only a small proportion of the overall genome size (Figure 1B).

**Table 3:** Genome annotation features of *Cordyceps guangdongensis* GD15

Feature	Total number	Total length (bp)	Average length (bp)	Length/ genome length (%)
gene	9,150	16,372,278	1,789.32	56.35
Exons	29,548	14,031,735	474.88	48.29
CDS	9,150	14,031,735	1,533.52	48.29
Introns	20,398	2,340,543	114.74	8.06
tRNA	111	9,519	85.75	0.03
rRNA	31	95,875	3,092.74	0.33
sRNA	121	7,369	60.9	0.025
snRNA	25	2,908	116.32	0.01
miRNA	26	1,766	67.92	0.006

**Figure 2:**
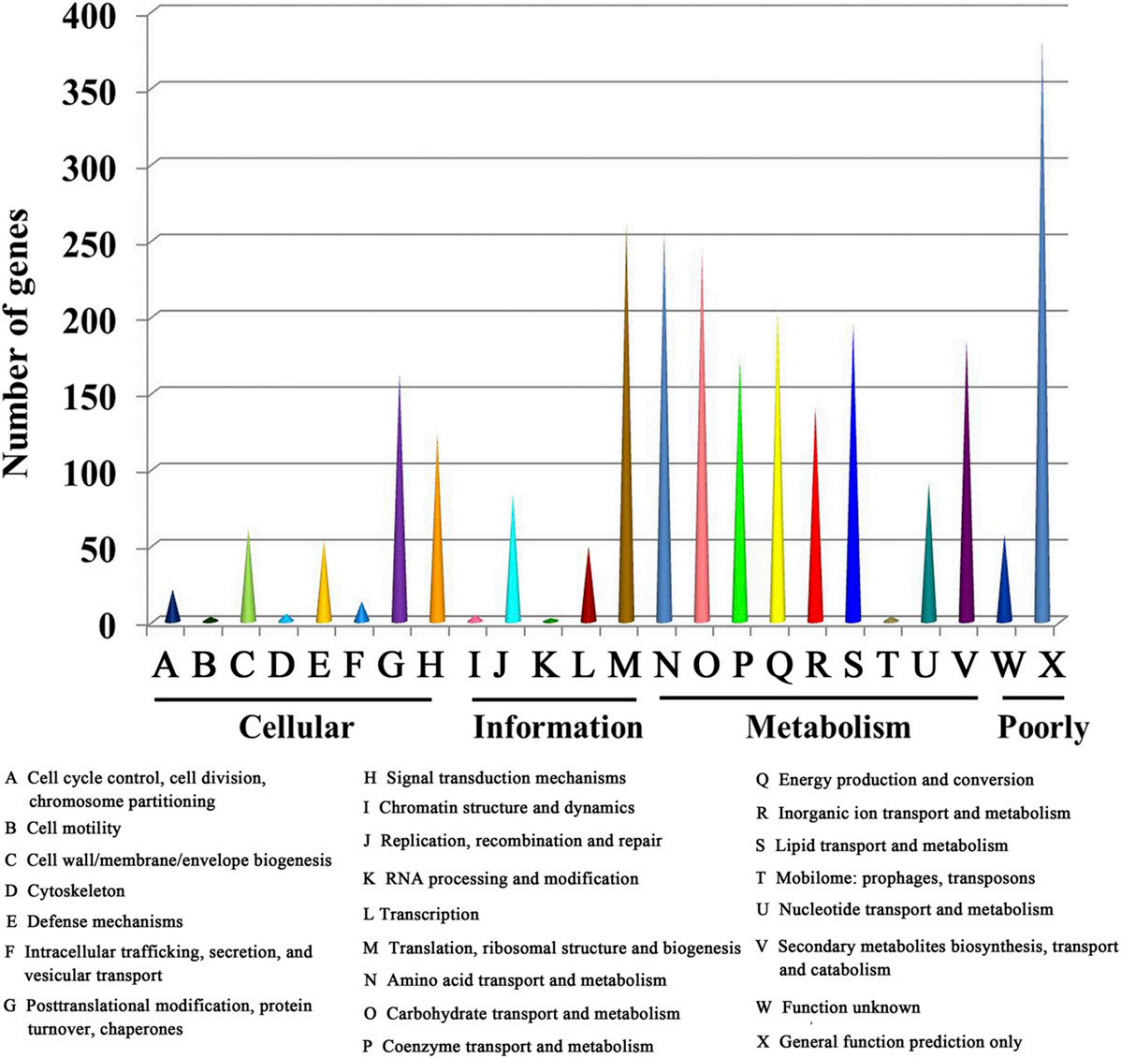
COG functional classification of proteins in the *Cordyceps guangdongensis* genome.

Of the 9150 identified genes, 8486 genes (92.74%) were annotated using the databases described in the methods section (Table S2). This present paper mainly focused on the genes involved in metabolic processes. Among all the genes predicted, approximately 48.90% (4475) were annotated by KEGG pathway, and in these genes, 15.57% (1425) of the total predicted genes were involved in metabolism accounted for the major proportion. Genes classified into functional categories based on the COG analysis accounted for 24.43% (2236), and in these genes, 12.01% (1099) of the total predicted genes were involved in metabolic processes, and approximately 2.01% (184) of the total predicted genes were related to the biosynthesis, transportation, and catabolism of secondary metabolites (Figure 2). The percentage of genes encoding CAZymes was 3.15% (289); these genes were contributed to substrate degradation processes in nutrition for fungal development and reproduction. Among the genes related to CAZymes, 103 genes encoding glycoside hydrolases (GHs) accounted for the largest proportion (1.12%) of the total predicted genes, followed by 78 genes encoding carbohydrate-binding modules (CBMs) (0.85%), and then 66 genes encoding glycosyl transferases (GTs) (0.72%). Genes acted as auxiliary activities (AAs) accounted for 0.32%. In addition, genes belonging to the carbohydrate esterases (CEs) and polysaccharide lyases (PLs) had much lower percentages of about 0.13% and 0.01% of the total predicted genes, respectively. Since the genes relevant to CAZymes in *Pleurotus eryngii* were not only involved in decomposition of organic materials, but also primordium differentiation and fruiting body development (Xie *et al.* 2018), the CAZymes genes identified in *C. guangdongensis* could likely be also involved in primordium differentiation and fruiting body development. These results are beneficial and provide the basis to further study the genetic and molecular mechanisms underlying fruiting body development.

### Repetitive elements

The cumulative sequences of repetitive elements identified in *C. guangdongensis* genome occupied 2.77% of the assembly sequences. The tandem repeats represented 1.42% of the genome assembly, with a total length of 412,989 bp; and the TEs represented 1.35% of the genome assembly with a total length of 393,608 bp. The total number of TE families analyzed with RepeatMasker in the genome assembly was 1534, of which 1527 (99.5%) belonged to the known TEs, including 1033 retrotransposons (Class I) and 494 DNA transposons (Class II); the remaining TEs could not be classified at the time of this study (Table 4 and Table S3).

**Table 4:** Transposable element repeat class analysis in *Cordyceps guangdongensis*

Repeat element family	Number of unique elements in family	cumulative length (bp)			% of genome assembly
**Class I - Retrotransposons**	**1,033**		**319,324**	**1.09898**	
** LINE**	343		57,004	0.19618	
L1	14		660		
others	329		56,344		
** LTR**	661		259,862	0.89435	
Copia	269		174,266		
DIRS	4		324		
ERV1	17		1,170		
Gypsy	314		79,093		
Pao	17		1,741		
others	40		3,268		
** SINE**	15		1,304	0.00448	
Alu	1		51		
Others	14		1,253		
** others**	14		1,154	0.00397	
**Class II - DNA Transposons**	**494**		**73,739**	**0.25378**	
CMC-EnSpm	44		3,001		
Dada	8		485		
Ginger	2		141		
hAT	4		440		
Merlin	3		108		
MULE-MuDR	29		4,628		
P	2		134		
PIF-Harbinger	11		729		
PiggyBac	63		19,337		
Sola	29		1,840		
TcMar-Tc1	14		3,780		
Zisupton	1		88		
others	284		39,028		
**Unclassified**	**7**		**545**	**0.00187**	
**Total**	**1,534**		**393,608**	**1.35466**	

Class I retrotransposons can be mainly divided into three groups of TEs, including LINE, LTR, and SINE; each group contains several subgroups. Retrotransposons, particularly L1, Copia, DIRS, ERV1, Gypsy, Pao, Alu, etc., are the easiest to be annotated; they are also the most abundant transposons in fungi (Suarez *et al.* 2018). Class II DNA transposons contain a lot of known groups and some unclassified members. Among the transposons, hAT, MULE, PIF-Harb and Tc1-Mariner were reported to be extraordinarily abundant in fungi, whereas transposons CMC and piggyBac have limited taxonomic distribution and seem to exist in only a few fungal taxa (Muszewska *et al.* 2017). Other transposons, including P, Sola, Dada, Ginger, Zisupton, and Merlin, had been identified only in a handful of species (Kojima and Jurka 2013; Iyer *et al.* 2014; Majorek *et al.* 2014).

Previous studies indicated that TEs contributed to genome size expansion and evolution (Cordaux and Batzer 2009; Sun *et al.* 2012) and played crucial roles in a wide range of biological events, including organism development (Kano *et al.* 2009; Garcia-Perez *et al.* 2016), regulation (Elbarbary *et al.* 2016) and differentiation (Morales-Hernández *et al.* 2016). In addition, they sometimes act as novel promoters to activate the transcription process (Faulkner *et al.* 2009; Mita and Boeke 2016). Therefore, the abundance in *C. guangdongensis* would be more significant in this regard; they should be noted and further studied for their application in fungal taxonomy and regulatory roles in fruiting body development.

### Transcription factors

**Figure 3:**
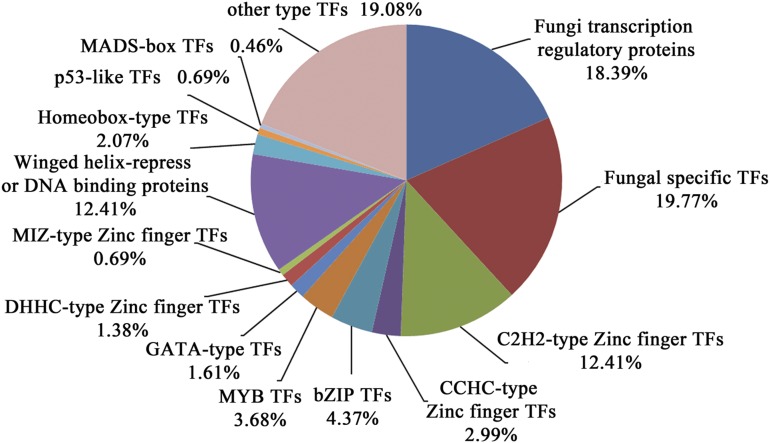
Transcription factors analysis in the *Cordyceps guangdongensis* genome.

Transcription factors (TFs) are essential for modulating diverse biological processes by regulating gene expression and playing central roles in organism development and evolution. In this study, functional annotation identified 435 genes of TFs in *C. guangdongensis*, accounting for 4.75% of the total predicted genes. Like other fungi, genes encoding fungal-specific TFs (86 members) and fungal transcription regulatory proteins (80 members) represented the two largest classes of TFs in *C. guangdongensis*, accounting for approximately 19.77% and 18.39% of the total predicted TFs, respectively; and followed by C_2_H_2_-type zinc finger TFs (54 members) and winged helix-repressor DNA binding proteins (54 members), accounting for approximately 12.41% and 12.41%, respectively (Figure 3).

Moreover, other different types of zinc finger TFs were identified, including 13 CCHC-type zinc finger TFs (2.98%), 6 DHHC-type TFs (0.06%) and 3 MIZ-type TFs (0.03%). Apart from these, there were 19 bZIP TFs (0.21%), 16 MYB TFs (0.17%), 7 GATA TFs (0.08%) and 9 homeobox-type TFs (0.10%). In *C. militaris*, majority of TFs, such as Zn_2_Cys6-type TFs, GATA-type TFs, bZIP TFs, and CHCC-type TFs, were differentially expressed during fruiting body developmental stages (Zheng *et al.* 2011). Hence, this information could help explore the regulatory mechanisms of TFs in fruiting body development in *C. guangdongensis*.

### Conclusion

High quality genome sequencing of *C. guangdongensis* was presented in this study. Two complete chromosomes and four single-end chromosomes were assembled through genome sequence analysis. In the genomic sequences, diverse transposable elements were identified, which may contribute to genome size and evolution. Moreover, transcription factors in the genome of *C. guangdongensis* were identified and classified; these transcription factors may facilitate further studies of fruiting body development. And above all, knowledge about the genome sequence of *C. guangdongensis* will reveal more detailed molecular information and facilitate further studies of fruiting body development and identification of secondary metabolites in *C. guangdongensis*.
